# Three layers in spinal canal

**DOI:** 10.1002/jgf2.638

**Published:** 2023-06-29

**Authors:** Satoshi Inaba, Hisatoshi Okumura, Ayaka Kobayashi, Atsushi Kawashima

**Affiliations:** ^1^ Division of General Internal Medicine Fukuchiyama City Hospital Kyoto Japan; ^2^ Department of Endocrinology and Metabolism Kyoto Prefectural University of Medicine, Graduate School of Medical Science Kyoto Japan

**Keywords:** Diagnostic Reasoning, Emergency Medicine, Hospital General Medicine, Internal Medicine

## Abstract

Contrast‐enhanced computed tomography revealed spontaneous spinal epidural hematoma, which mimicked aortic dissection.

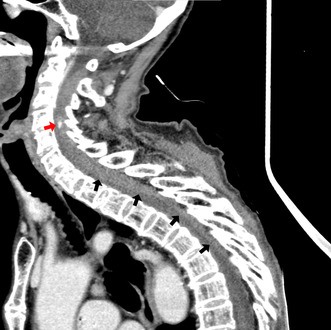

A 90‐year‐old woman with a history of hypertension presented to the emergency department at night with a 3‐h history of sudden neck‐to‐back pain, followed by loss of sensory, motor, and bladder function. She had no prior history of injury or anticoagulant medication use.

At admission, her vital signs were as follows: blood pressure, 210/80 mmHg without any difference in the right and left arms; pulse rate, 48 beats/min; respiratory rate, 20 breaths/min; temperature, 36.1°C; and oxygen saturation, 100% on ambient air. She remained lucid throughout her stay. General physical examination findings were normal. Neurological examination demonstrated right‐dominant paresis, with manual muscle testing (right/left) indicating biceps of 2/4 and iliopsoas of 2/3. The patient also exhibited right‐dominant dysesthesia in the distal upper and lower extremities but no thermal hypoalgesia was observed. Deep tendon reflex testing indicated an increased left biceps reflex. Cerebral neurological findings were normal.

Blood tests showed an elevated D‐dimmer level of 2.9 μg/mL (reference range <1.0) but other laboratory data were within normal limits.

Suspecting aortic dissection (AD), a night‐shift doctor performed an urgent contrast‐enhanced computed tomography (CT), which did not reveal a false lumen. Although the cause of her symptoms was unknown, she was admitted for follow‐up.

On the following day, the patient's symptoms persisted without improvement. The hospitalist, who had taken over the patient's care from the previous on‐duty physician, reviewed the previous day's CT scan. In a reconstructed sagittal view, an epidural space‐occupying lesion with extravasation at the C4 level was identified (Figure 1), which suggested a diagnosis of spinal epidural hematoma (SSEH). To confirm the diagnosis, magnetic resonance imaging (MRI) was performed.

**FIGURE 1:**
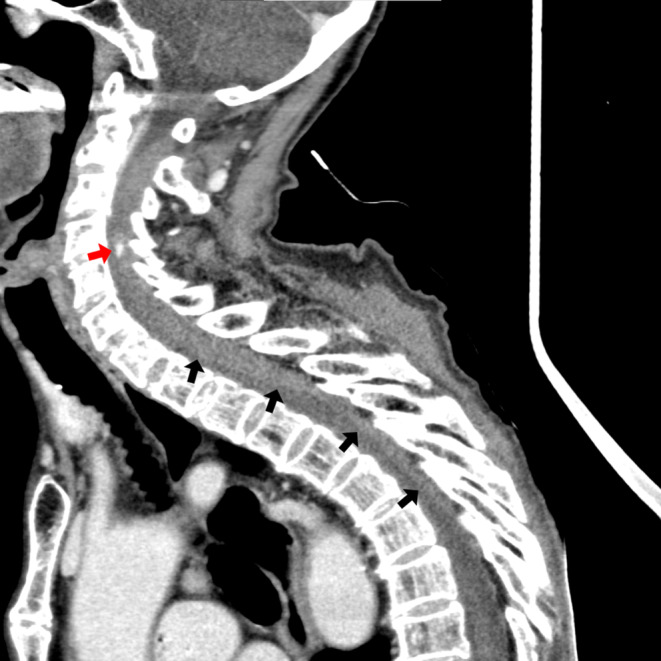
Sagittal view of a contrast‐enhanced CT scan revealed an epidural space‐occupying lesion with extravasation (red arrow) that has a clear boundary with the low‐absorption area (black arrows).

T2‐weighted imaging (T2WI) of MRI also revealed a three‐layered structure from the C4 to T6 level in spinal canal, consisting of the spinal cord (hypo‐intense), spinal fluid (hyper‐intense), and hematoma (iso‐intense) from the ventral, which was consistent with the diagnosis of SSEH (Figure 2).

**FIGURE 2:**
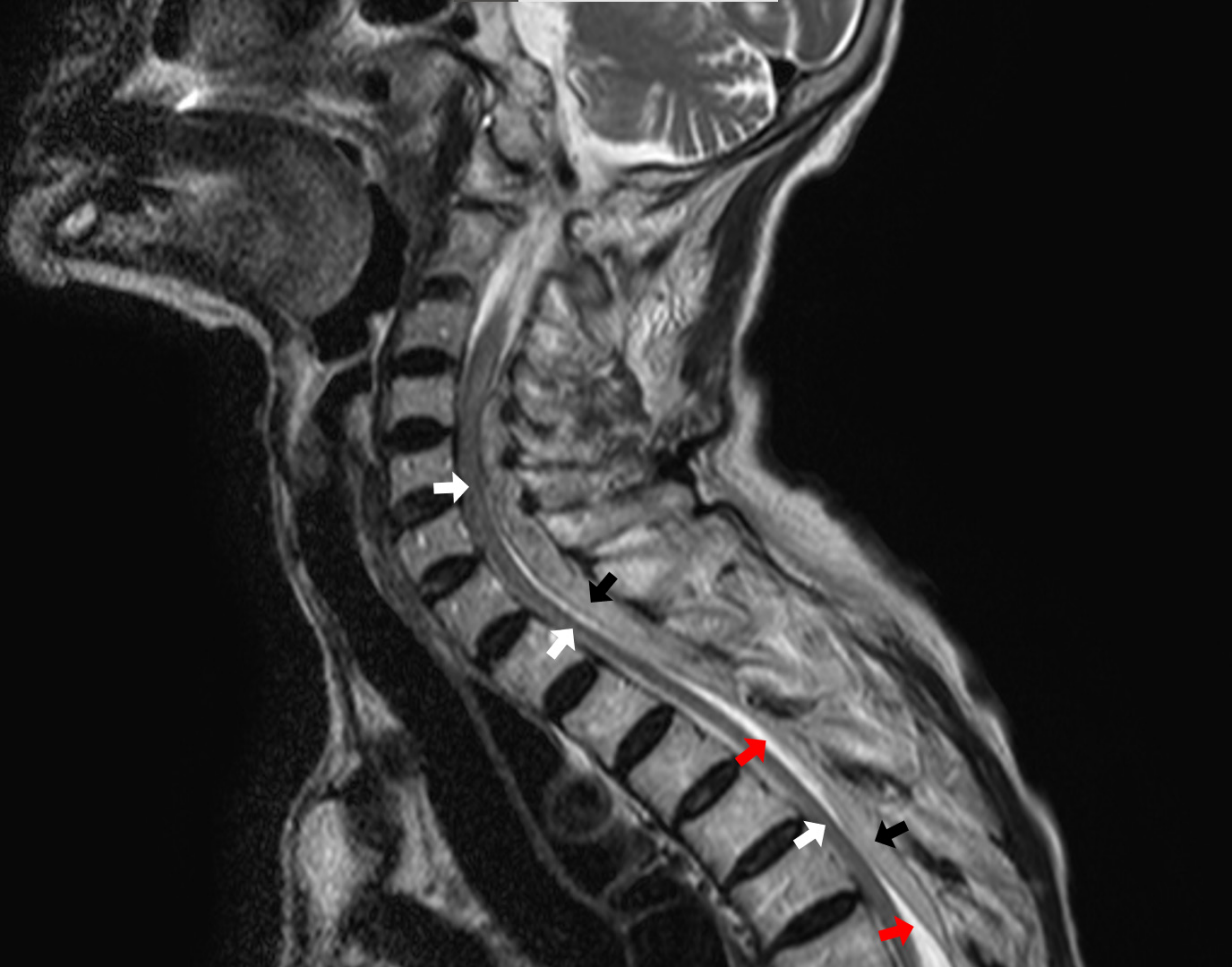
Sagital view on T2‐weighted imaging of MRI revealed a three‐layered structure in the spinal canal, consisting of the spinal cord (white arrows), spinal fluid (red arrows), and hematoma (black arrows).

Despite the delay in diagnosis, a laminectomy and blood drainage were performed within 24 hours of onset and her symptoms largely resolved.

SSEH is a rare but serious and treatable disease that presents with sudden neck‐to‐back pain and progressive paralysis.^1^, ^2^ Its peak incidence is in the 60s, and it is almost evenly distributed between genders.^1^ Hypertension and anticoagulant therapy have been identified as frequent risk factors but the cause remains unknown in approximately half of the cases.^2^ SSEH is typically diagnosed by MRI but it can also be detected on contrast‐enhanced CT, as in this case.

The sudden onset of pain and paralysis in SSEH can mimic the presentation of AD or stroke,^3^, ^4^ leading to delayed or incorrect diagnosis. When there are still some inconsistencies in neurological findings after a CT scan suspected of AD has found no dissection, the spinal canal should be examined.

## CONFLICT OF INTEREST STATEMENT

The authors have stated explicitly that there are no conflicts of interest in connection with this article.

## PATIENT CONSENT STATEMENT

The authors have obtained patient consent.
